# Touching the unconscious in the unconscious – hypnotic communication with unconscious patients

**DOI:** 10.3389/fpsyg.2024.1389449

**Published:** 2024-06-20

**Authors:** Ernil Hansen

**Affiliations:** Department of Anesthesiology, University Hospital Regensburg, Regensburg, Germany

**Keywords:** hypnosis, coma, resuscitation, general anesthesia, communication, intensive care, unconsciousness, suggestions

## Abstract

If hypnosis means contact to the unconscious to modulate psychological and physiological functions by means of suggestions, and if this is facilitated by attenuation of the critical mind, then the question arises as to whether suggestions also have an effect when waking consciousness is otherwise eliminated, namely by coma or anesthesia. A prerequisite would be perception, which actually is evidenced by reports of patients after traumatic brain injury, artificial coma, resuscitation or general anesthesia. Moreover, posttraumatic stress disorder (PTSD) frequently observed after these medical situations is hardly explainable without some sort of awareness under such conditions. Even advanced neurophysiological diagnostic cannot yet rule out consciousness or sensory processing. Especially reference to perception during unconsciousness is given by the results of a recent multicenter study on the effects of hypnotic communication with patients under controlled adequate deep general anesthesia. The observed reductions in incidence and severity of postoperative pain, opioid use, nausea and vomiting cannot be explained by the reaction of a few but only by a considerable proportion of patients. This leads to a strong plea for a more careful treatment of unconscious patients in the emergency room, operating theater or intensive care unit, for the abandonment of the restriction of therapeutic communication to awake patients, and for new aspects of communication and hypnosis research. Obviously, loss of consciousness does not protect against psychological injury, and continuation of communication is needed. But how and what to talk to unconscious patients? Generally addressing the unconscious mind with suggestions that generally exert their effects unconsciously, hypnotic communication appears to be the adequate language. Especially addressing meaningful topics, as derived from the basic psychological needs and known stressors, appears essential. With respect to negative effects by negative or missing communication or to the proposed protective and supporting effects of therapeutic communication with patients clinically rated as unconscious, the role of consciousness is secondary. For the effects of perceived signals and suggestions it does not matter whether consciousness is absent, or partial, or unrecognized present.

## 1 Introduction

Various conditions can lead to an alteration or even loss of consciousness. The origin can be physiological processes like sedatives, impairment of the brain by drugs (psychedelics, alcohol, sedatives, narcotics), or metabolic, ischemic or traumatic brain injury (Young, 2009; Eapen et al., 2017). With increasing severity this becomes a medical issue, particularly in the form of general anesthesia or coma. The extent can be scored according to residual responsiveness, e.g., by Glasgow Coma Scale, Ramsay-Scale, or Sedation-Agitation-Scale (Bordini et al., 2010). With total unconsciousness defined as the lack of any reaction to external stimuli, communication usually comes to an end, both from the patient’s and the helpers` side. But is this really so, and is it reasonable? Does unresponsiveness exclude perception? In case of doubt, communication should not be discontinued.

Several observations of emergency, surgical or intensive care patients suggest perception even in unconscious patients. When hypnosis is the establishment of contact with the unconscious and the influencing of psychological and physiological functions by means of suggestions (Erickson, 2009; Elkins et al., 2015; Linden et al., 2024), and if the possibility for this is opened up primarily by bypassing waking consciousness and the elimination of critical reason (Ahlskog, 2018; Peter, 2024, this issue), then the question arises as to whether hypnotic suggestions also have an effect when waking consciousness is otherwise eliminated, namely by coma or general anesthesia. Touching the unconscious in the unconscious.

Considering these situations and conditions it is important to remember that perception and its impact largely depend on the importance and meaning of the perceived signal or message. Moreover, suggestibility, i.e., the extend of the reactions to suggestions, is massively increased in trance, a non-ordinary state of consciousness that is induced by hypnosis or extreme situations (Peter, 2024, this issue). The events that lead to the states to be discussed, namely an accident or a trauma resulting in coma, the need for surgery under general anesthesia, or complications and disorders making intensive care necessary, all represent such “extreme,” trance-inducing conditions. Could the triggered elevation in suggestibility also be significant when in the course of such events unconsciousness has occurred?

## 2 Consciousness/unconsciousness

Before evaluating and discussing evidence for perception in different states and disorders of consciousness (DoC) and the possibility of communication, some definitions or rather what is understood by this in the following seem appropriate. “Consciousness” is a subjective experience that plays a considerable role in the psychological, physical, and behavioral reactions to external and internal stimuli. However, the precise definition can vary considerably between philosophers, neurophysiologists and clinicians. Here, clinical aspects are of priority. “Connectiveness” refers to the connection of consciousness to the external world allowing experience of external stimuli (Sanders et al., 2012). Examples of disconnected consciousness are dreaming in sleep, namely rapid-eye-movement (REM) sleep, or dreaming in anesthesia. It also can be induced by hypnosis. “Memory” is not essential for experience, nor is it for consequences of perception. For recalls of events that have taken place during unconsciousness a distinction is made between explicit and implicit memory. Explicit memories are reported spontaneously by the awakened patient, or can be determined by structured interviews or questionnaires after the phase of unconsciousness. Implicit memories are not consciously remembered by the patient, but can be evaluated under hypnosis (Levinson, 1965; Cheek, 1966), or by association techniques (Schwender et al., 1994). Remembrance, however, is strongly dependent on attention, emotional content and meaning. “Responsiveness” means the behavioral interaction with the outside world, and is divided in spontaneous and goal-directed (following a command) responses. This responsiveness is not only dependent on the perception of an input, but also limited by restrictions in the output, for instance by pharmacological muscle relaxation, psychic or neurologic paralysis, attention, and motivation. Another indicator for perception in unconscious patients is the phenomenon of near-death-experiences (NDE). They can be described as internal awareness experienced in unresponsive conditions and classified as disconnected consciousness (Martial et al., 2020). Such memories are reported after situations close to death, e.g., cardiac arrest or coma, characteristically connected with unconsciousness. The reported memories can be detailed and true, or false (Martial et al., 2018). Both harbor the risk of traumatization, e.g., the true perception of fixation straps during intensive care or the oneiroid “false memory” of being a war prisoner.

Clinically, mainly behavioral responses and memories are used for assessment of consciousness, which both neither allow precise judgment of consciousness, nor evaluation of the consequences of any perception. Even fragments of information can be behaviorally functional yet kept out of consciousness (Mashour, 2013).

Considering the effects of external stimuli including communication on patients that present as “unconscious,” one has to deal with all these components in its different appearance and characteristics, and their combinations. This creates great complexity and hampers simple equations like unresponsiveness = unconsciousness (Sanders et al., 2012). Moreover, this complexity is the reason for many disadvantageous misconceptions in a number of severe medical situations (Table 1).

**Table 1:** Frequent disadvantageous misconceptions with unconsciousness.

Perception and awareness only in consciousness
Unresponsiveness excludes perception
Perception and traumatization avoided by sedation
Even if some awareness, effects of stimuli are attenuated in unconscious patients
Unconscious patients need no communication
Suggestions and communication less effective in unconscious patients

**Figure 1:**
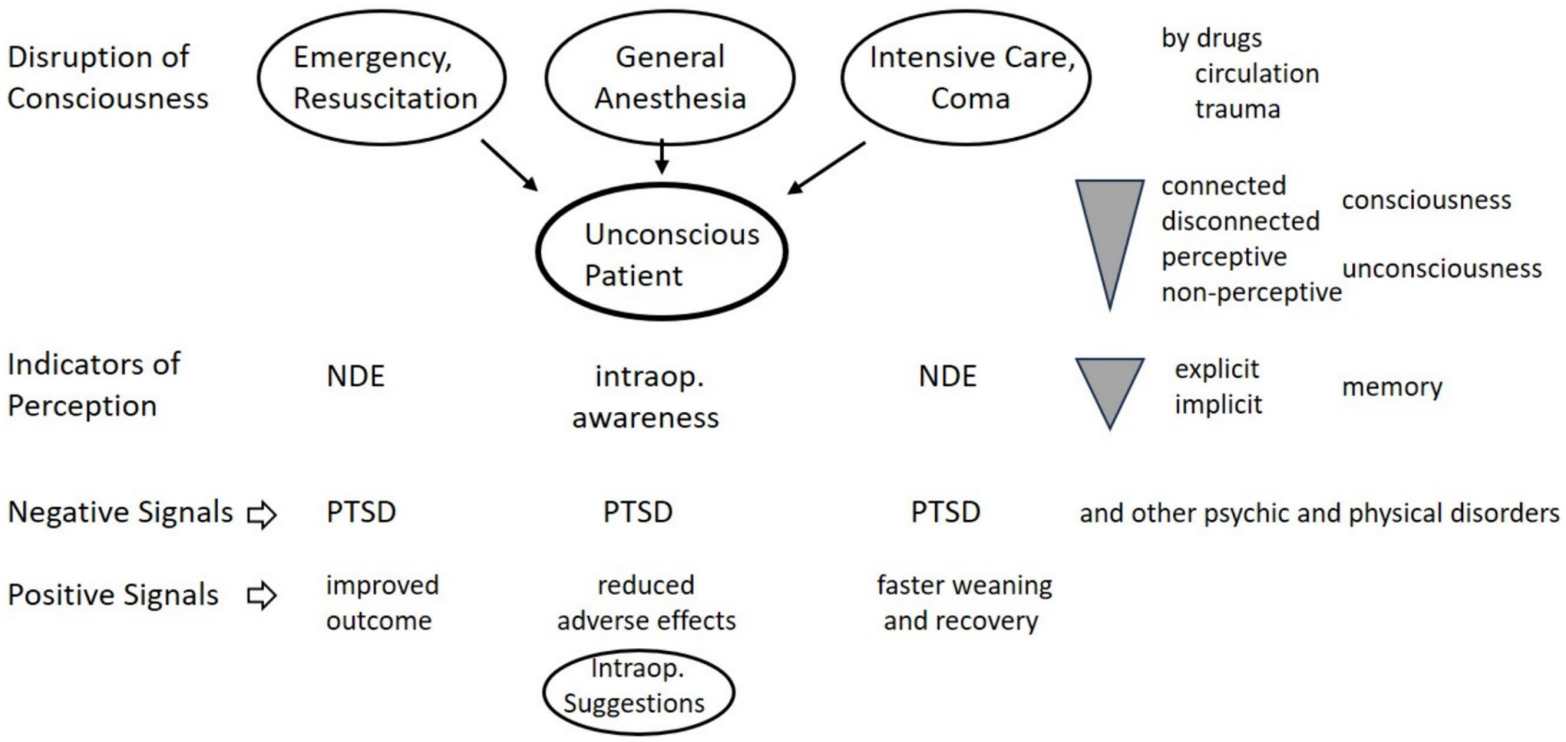
Unconsciousness and perception. NDE, near death experience; PTSD, posttraumatic stress disorder.

The “cognitive unbinding paradigm” is based on the “integrated information” theory that describes unconsciousness as interrupted information. Consciousness is lost due to impaired communication across brain networks and the consequent isolation of cognitive processing modules (Mashour, 2013). Thus, isolation rather than extinction of neural activity or sensory processing is sufficient for unconsciousness. Or other way around, areas and networks in the brain involved in information synthesis and inter-modal processing may be disrupted, while sensory networks and processing can persist despite unconsciousness. Moreover, from hypnosis we know that psychological and physiological functions are especially regulated and modifiable in the unconscious mind, and reaching the conscious level is not necessary for effects of verbal and nonverbal suggestions (Knafo and Weinberger, 2024; Peter, 2024, this issue). Specific rather than general neural network disruption is the common cause of unconsciousness, with differing resilience of brain areas and their connections affected by trauma, circulatory disorder or drugs. Neuroimaging has demonstrated persistent sensory processing during impaired network communication in coma or general anesthesia. Functional connectivity of sensory networks was found relatively unperturbed for instance after anesthetics (Mashour, 2013). Cognitive processing can persist in unconscious states, while the binding of this activity into a meaningful conscious representation is inhibited, which on the other hand is not required to trigger effects. A summary of connections between consciousness and perception are depicted in Figure 1.

## 3 Emergency medicine and resuscitation

One of the most exciting reports of positive communication in emergency medicine is the “Kansas experiment.” There, avoidance of unrelated or negative conversation and a positive text recited during transport to the hospital resulted in more patients surviving the transport and the hospital stay, and quicker recovery rates (Jacobs, 1991). The hypnotherapist M.E. Wright had trained three groups of ambulance attendants to do so for 6 month and compared outcome to control groups. In this study, the text (Table 2) was used for accident victims regardless “whether they were stuporous, conscious, or unconscious,” which means that unconscious patients were included. It was assumed that with the trauma “a narrowing of the total psychological functioning has occurred so that there is an acute responsiveness in some areas and a lack of awareness in others,” and that “shock can be considered a radical mobilization of the body to preserve essential life functions to sustain survival” maintaining minimal reception of information. Wright had the idea that “in such situations the person’s usual critical responsiveness to the environment has been altered so that whatever stimuli do reach are often subject to literal translation and can either aggravate or support the life systems that are hanging on...” This description also perfectly applies to other forms of unconsciousness (see the following) and to states of “natural trance” induced by stress, fear and pain in emergencies or when facing surgery (Cheek, 1962a). It is a pity and a shame that in the time after the “Kansas experiment” of 1976, this study was never reproduced in the subsequent 48 years to be published in a medical journal.

**Table 2:** Text of the “Kansas Experiment” read out during transport of accident victims (Jacobs, 1991).

The worst is over. We are taking you to the hospital. Everything is being made ready. Let your body concentrate on repairing itself and feeling secure. Let your heart, your blood vessels, everything, bring themselves into a state of preserving your life. Bleed just enough so as to cleanse the wound, and let the blood vessels close down so that your life is preserved. Your body weight, your body heat, everything, is being maintained. Things are being made ready at the hospital for you. We’re getting there as quickly and safely as possible. You are now in a safe position. The worst is over.”

The most important and convincing evidence for perception in unconscious emergency patients stems from studies on cardiopulmonary resuscitations (CPR). With his book “Life after life” of 1975 based on 150 interviews, Raymond Moody shaped the expression “near-death experience” and raised the public and scientific interest in this topic (Moody, 2001). In 1979 a study was published on 2000 patients interviewed after life-threatening situations. 60% reported near-death-experiences (NDE) including perception of the external processes and the stress of not being able to make themselves heard (Shoonmaker, 1979). In addition to the selective evaluation of patients in appropriate situations, there exist also epidemiological studies using representative surveys. NDE were found in 15% of US Americans, with one third reporting extraordinary experiences including out-of-body-experience (OBE), which usually is combined with perception and description of the external events. A survey of 4,000 Germans revealed NDE in 4.5% in the normal population, mainly after experiencing emergencies, surgeries or cardiac infarctions, with 6% actually suffering clinical death (Knoblauch et al., 2001). Particularly noteworthy is that 65% of them felt mentally wide awake, and 30.5% described OBEs.

More precise with regard to perception during death and CPR is the report of van Lommel et al. (2001). In a prospective study of 344 patients surviving hospital resuscitation after cardiac arrest, 18% had NDE, with no correlation to oxygen deficiency, duration of cardiac arrest and massage, medication, or religious belief. In some of these patients a flat EEG was recorded. 25% of the patients had an OBE and reported details of the CPR. One patient told the nurse exactly where she had placed his dental prosthesis lost during resuscitation. In another study, 90 out of 93 reports by patients after OBE were accurate (Holden et al., 2009). In 2014 the results of the AWARE (AWAREness during REsuscitation) study, a prospective study on awareness during CPR, was published (Parnia et al., 2014). Of 140 patients surviving in-hospital cardiac arrest and resuscitation (16% overall survival rate), 9% had NDE, while 2% described awareness with explicit recall of actual events related to their resuscitation, including seeing and hearing the rescuers. One had a verifiable period of conscious awareness “during which time cerebral function was not expected...” In the second prospective AWARE study 11 of 28 (21%) surviving and surveyable patients after resuscitation for cardiac arrest reported memories and perception from cardiac arrest without external signs of consciousness (Parnia et al., 2023). One described “they were putting two electrodes to my chest, and I remember the shock.”

All of these patients had suffered clinical death defined as the period of unconsciousness caused by total lack of oxygen in the brain (anoxia) resulting from the arrest of circulation, breathing, or both. Under the subsequent conditions of cardiac low flow during manual cardiac compressions the brain can survive but not function (van Lommel, 2011). It has been argued that effective CPR could allow temporary awareness. This is discrepant to the fact that even during effective CPR cardiac output and oxygenation are impaired and limited, which is incompatible with higher cerebral performances. The mentioned reports contrast completely to CPR-induced consciousness with observable signs, observed in 0.2–0.9% of resuscitations (West et al., 2022). On the other hand, an important characteristic of NDE is the high alertness and awareness reported by the patients, as well as the exceptionally good recall of the perceived experiences, even decades later. This reflects non-congruence of consciousness and perception. Furthermore, reports of synchronized gamma oscillations - signifying heightened lucid consciousness - in humans and animals on electroencephalography (EEG) during cardiac arrest and death, has raised the intriguing possibility of electrocortical biomarkers of lucid/heightened consciousness during cardiac arrest (Borjigin et al., 2013).

Another clue to perception could be the occurrence of posttraumatic stress disorder (PTSD) or other newly developed psychiatric morbidity after CPR such as depression, anxiety disorder, or substance abuse (Oh et al., 2022). How can somebody be stressed without being aware of the stressful situation? A high incidence of PTSD following CPR of 27% was reported (Gamper et al., 2004). Interestingly, sedation during CRP does not prevent PTSD but worsens survival.

## 4 Coma and intensive care

Consciousness quite often is impaired in patients during intensive care due to coma after traumatic or ischemic brain injury, intoxication, or infectious or metabolic disorders, or due to pharmacological coma ranging from sedation to “medically-induced coma” (to reduce brain metabolism). The resulting disorders of consciousness (DoC) have a diverse appearance including coma, all sharing unresponsiveness (Hannawi et al., 2015). Coma is defined as a state of profound unawareness from which the closed-eyes, non-communicating patients cannot be aroused (Kondziella et al., 2020). In contrast, the term “vegetative state/unresponsive wakeful syndrome” denotes a condition of wakefulness without awareness, where patients open-eyes exhibit only reflex behaviors. These patients may recover to a “minimal conscious state” (MCS), where non-reflex cortically mediated behaviors fluctuate spontaneously or dependent on certain stimuli or specific situations. In addition, assessment of consciousness perception may be further obscured by existence of islands of consciousness and functional fluctuations. The situation is aggravated by an apparently innate reflex to stop communication when someone has their eyes closed.

The prevalence of PTSD after intensive care including such patients is high, amounting to 20-25% (Parker et al., 2015). The strains are manyfold: being fixed and restrained in bed, treated with vasopressors (that mimic stress response) or paralyzed, continuous noise and lighting, unpleasant manipulations, often mechanical ventilation, and the perceived severity of the life-threatening illness itself (Warlan and Howland, 2015). These strains are not restricted to conscious perception. For instance, PTSD signs and symptoms are found in 35% of mechanically ventilated patients that usually are comatose or sedated (Bienvenu et al., 2013). Rather than the clear memories of an awake patient, these are the delusional memories of frightening perceptual experiences that are associated with the development of PTSD, and are more likely to be retained over time (Jones et al., 2007). However, having no memory of ICU is not beneficial either (Granja et al., 2008). No memory of their admission to the ICU in half of the patients was found strongly associated with the development of PTSD. A considerable portion of these patients is sedated or unconscious at the time of admission, an important cause for their amnesia for that time. Moreover, the use of restrains, necessitated by unconscious agitation and movements, is associated with PTSD (Davydow et al., 2008).

The idea that psychological trauma is dependent on conscious perception and that sedation would reduce risk for PTSD has turned out to be wrong. In fact, the risk is enhanced by sedation. A review found use of benzodiazepines and duration of sedation correlating with PTSD (Wade et al., 2013). Possible explanations are that conscious perception reduces the trauma, or that the trauma is aggravated as communication is often stopped as soon as the patient’s eyes are closed, and he or she is ignored while inappropriate conversations may occur. The realization that drug-induced loss of consciousness is not protective, and can even enhance stress and PTSD led to attempts to reduce the stress of mechanical ventilation by lighter levels of sedation, intermittent spontaneous breathing trials and early extubation. However, their validation is yet missing. Instead, hypnotic communication has been used successfully to reduce stress and fasten weaning from the respirator (Szilágyi et al., 2014). Most suggestions, both negative and positive ones, especially those of company and care, are transmitted to and perceived by the unconscious mind. An example is the calming down of heart rate when a visiting relative speaks to a deeply sedated patient. Patients may be able to unconsciously gauge a nurse’s or doctor’s intention and sense, if they are stressed or compassionate. Difficult to frame into a study, most intensive care physicians can recall patients that after long recovery report events and words from times where they were considered unable to hear or perceive anything. Among other things, discussions and decisions are reported about to stop artificial coma (used to reduce brain metabolism during restricted blood flow with increased intracranial pressure) or to use extracorporal membrane oxygenation (ECMO, “artificial lung”). At the time of such decisions patients usually are deeply unconscious. Likewise, NDE have also been reported after severe brain injury of traumatic or other origin (Hou et al., 2013). Incidence was reported with 15%, and correlation to mechanical ventilation, sedation, surgical reason for admission, and dissociative propensity (Rousseau et al., 2023).

Severe acquired brain injuries resulting in a DoC provide a model from which insights into consciousness can be drawn (Di Perri et al., 2014). Diagnosis is difficult when based only on behavioral assessments, common in clinical routine. Latest research in terms of both improving the diagnosis of patients with DoC, and understanding the brain processes underlining consciousness, reveals an underlying broad and more complex than previously thought alteration of brain connectivity architecture. However, neuroimaging and electrophysical techniques are still insufficient to detect possible consciousness residuals in severely traumatic brain injured patients.

## 5 General anesthesia

Interestingly, general anesthesia is also called drug-induced hypnosis, and literature searches for the term “hypnosis” regularly yield references about anesthesia. But can patients under anesthesia perceive anything at all, especially words? There are indications for it. David Cheek, an American gynecologist and hypnotherapist, was the first to point this out after narrations of his patients under hypnosis of events and conversations from earlier operations (Cheek, 1962b), without being believed. However, reports of intraoperative wakefulness with explicit memory increased, and to this day it occurs in about 0.2% of anesthetics (Ghoneim, 2000; Mashour and Avidan, 2015). Implicit memory can be revealed by association or under hypnosis. For example, in the 1990s Agnes Kaiser incorporated a text around the Robinson Crusoe story into a study using acoustic-evoked potentials (AEP) to investigate the influence of different anesthetic procedures on the primary auditory pathway as a way to measure depth of anesthesia (Schwender et al., 1994). The text played intraoperatively led postoperatively to associations Friday-holiday or Friday-island or Friday-Robinson instead of Friday-weekend-beginning. Effects of the positive text were unfortunately not studied at all. The observed high incidence is now considered to be due to inadequate anesthesia, and the actual incidence of implicit recall is reported to be 2%. Due to the negative content and consequences of such intraoperative perceptions, including a high incidence of PTSD, these are usually attributed to “inadequate anesthesia” and every effort is being made to avoid it. Modified anesthesia management and anesthesia depth monitoring have indeed reduced the traumatizing occurrence of intraoperative wakefulness, but has not yet been able to eliminate it (Tasbihgou et al., 2018). Moreover, primary sensory areas are relatively resistant to loss of consciousness under anesthesia (Nourski et al., 2018).

However, the recall of memories is shaped by meaning. What is important enough of intraoperative auditory stimuli to be remembered? A much higher incidence of intraoperative awareness was clearly demonstrated in the experiments of B.W. Levinson in South Africa in the 1960s, which today would be considered unethical (Levinson, 1965). In 10 patients under EEG-controlled general anesthesia a hypoxia alarm was simulated intraoperatively. “He′s got blue lips! There are ventilation difficulties...” and “I do not like this color!.” Postoperatively, four of the patients under hypnosis repeated the words correctly, while another four showed a fear reaction with termination of the trance. Accordingly, incidence of perception was 80%, in striking contrast to the otherwise reported occurrence of intraoperative awareness. Presumably, the reason for the high incidence in this case is the high, life-threatening significance of the given suggestion. Interestingly, massive efforts to rule out “intraoperative awareness” by EEG-derived monitors of anesthetic depth were not successful. The incidence can be reduced but not eliminated, its existence is not limited to insufficient anesthesia.

On the other hand, the phenomenon of intraoperative perception has repeatedly led to attempts to use it for positive suggestion. Some studies have reported reductions in pain, anxiety, postoperative nausea and vomiting (PONV), and subsequent need for medication (Williams et al., 1994; Nilsson et al., 2001). A recent meta-analysis identified 32 adequate randomized controlled trials (RCTs) from 7,427 reports involving 2,102 patients, but showed no effects on pain intensity or psychological distress, but small but significant positive effects on recovery and use of medication (Rosendahl et al., 2016). These findings raised hope that a non-pharmacologic approach such as therapeutic suggestion during general anesthesia might be beneficial for surgical patients. However, the RCTs identified were relatively old (1986-2001), small in size, and heterogeneous in design. In addition, therapeutic and prophylactic regimens have changed in the intervening period, the management and depth of anesthesia were not standardized in these studies, and the suggestions used were heterogeneous and often included negations. The approach also did not find its way into clinical routine anywhere.

However, recently the effect of hypnotic suggestions was investigated again. A controlled, randomized, triple-blinded multicenter study was conducted at five German university hospitals with 385 patients undergoing painful surgery of 1–3 h duration under general anesthesia (Nowak et al., 2020, 2022). A 20-min text set to background music, followed by a 10-min break, was played repeatedly over earphones for the entire duration of the surgery, and a text on anesthesia withdrawal was played in the final phase. The depth of anesthesia was strictly controlled and the intervention strictly during anesthesia only. The control group also received earphones but no audio recording. The intervention text was based on hypnotherapeutic principles, and did not contain negations (such as “they will not be in pain!”). Especially, issues of meaning such as competence and caring of the surgical and anesthesiologic team, self-regulation, dissociation to a safe place, affirmation, fear control, and confidence were addressed (Table 3) (Link to the text and audio file: https://www.frontiersin.org/articles/10.3389/fpsyg.2022.898326/full#supplementary-material). The results were a significant reduction in postoperative pain level (NRS) by 25% over the 24-h observation period. In line with this, a significantly reduced need for analgesics, namely the opioid piritramide and also the additional medication with non-opioids, by one third was observed. With 36.6% patients without any analgesics a number needed to treat (NNT) of six was found. This means that if six patients received this intraoperative communication, postoperative pain medication (including its potential side effects) was avoided entirely in one patient as a result of this treatment alone (Nowak et al., 2020). In these patients at increased risk for postoperative nausea and with vomiting (PONV), that common and debilitating side effect of surgery and anesthesia also was significantly reduced with the hypnotic intervention. Incidence of both early and late manifestations, i.e., early and delayed PONV, were halved. Moreover, an observed NNT of 7 indicates that medication with antiemetics can be avoided entirely in 1 of 7 patients (Nowak et al., 2022). This study demonstrates high efficacy in reducing side effects of anesthesia and surgery with a simple, practical, non-pharmacological intervention. In addition, it makes the case for a wide application of intraoperative hypnotic suggestions, as well as perioperative therapeutic communication for surgical patients in general.

**Table 3:** Text example from study on intraoperative suggestions (Nowak et al., 2020).

“You are sleeping sound and deep. And you can relax and rest, recover and draw strength, because you are safe now, well-protected. Everything that you hear and see and feel contributes to your best care. And that’s why you can completely concentrate on your body’s own way to heal itself.” …
“This consisting beeping sounds of the monitor shows your smooth, rhythmic heartbeat. Your blood pressure is strong and steady. The most essential tasks you are performing yourself, organ perfusion, blood coagulation, immune defence, and many more. We healthcare guides just pay attention and care so that you and your body find optimal conditions. As your mind is resting your body can concentrate fully on self-healing and self-protection. All of your organs, your heart and your blood vessels, are working together to ensure wellbeing, safety and healing.” …

## 6 Discussion

### 6.1 Significance for medicine

There is another point worthy of consideration: The results of this study cannot be explained by the known “intraoperative awareness” with the reaction of only a few patients, but suggest that a considerable portion of patients can perceive auditory signals and suggestions under general anesthesia. Moreover, insufficient depth of anesthesia was excluded in this trial, in contrast to former older studies on intraoperative suggestions (reviewed in Rosendahl et al., 2016). Therefore, given these results and other evidence provided above that patients might be traumatized during unconsciousness, namely during resuscitation, general anesthesia or coma, we are faced with the fact that these patients are not shielded from perception. Their experience may include negative, disturbing and harmful words, noises, or sensations (Hansen and Zech, 2019). However, the same channel could be used for positive, helpful, healing suggestions.

“BE CAREFUL, THE PATIENT IS LISTENING should be engraved over the door of every operating room, every recovery room, every intensive care unit in every hospital” stated David Cheek 58 years ago, when he was first to describe a phenomenon meanwhile known as “intraoperative awareness” (Cheek, 1966). Nevertheless, the practice in operating theaters or intensive care units has not changed to a consistent considerate wording. Intensive care nurses may disagree and say that they now do announce their interventions: “We will turn you on your side.” “Do not be frightened, we will wash you now.” But such informative announcements miss a helpful meaning. Only with a supportive, meaningful message for the patient does such conversation becomes therapeutic with effects on health and healing. More appropriate statements would be: “We’ll turn you on your side for your comfort.,” “We will wash you now to keep you clean and to support your healing.,” “This temporary fixation in bed is for your safety.” Not just information and usual talking is needed, but “Therapeutic Communication” that has an impact on psychological and physical functions and thereby on symptoms, illness, healing, and well-being of the patient (Hansen, 2024). That is why it is now time for a new call (Hansen, 2022).

Half the challenge would be addressed and solved if we could stop or at least contain negative suggestions and nocebo-effects that are omnipresent in medicine (Hansen and Zech, 2019). Accordingly, avoidance or reduction of the negative influences are mandatory for all patients, the more for patients suffering acute disorders of consciousness. We know from hypnosis that suggestions, in general, do not act on a conscious level but reach the unconscious mind to exert their effects (Peter, 2024, this issue). Similarly, non-conscious activation of placebo and nocebo responses has been demonstrated (Jensen et al., 2012). Consciousness is no prerequisite for perception and subsequent psychological and physical reactions. Subliminal stimuli, masked from conscious awareness, are known to modify behavior, and the amygdala can be activated in the absence of cortical processing (Ohman et al., 2007). Moreover, there is evidence for unconscious learning (Clark et al., 2002). As a consequence, careful handling of unconscious patients is warranted. Even more: Those who are not convinced of the existence of perception by the unconscious should at least accept some kind of “reversal of the burden of proof.” They should ask themselves: What would I want to experience or what would I want to hear, if I were unconscious and there was the slightest chance that I might experience anything after all? When in doubt give the benefit of the doubt to the unconscious but perceiving patient!

The idea that even if there is some awareness in the unconscious patient, the brain will be attenuated and effects of signals from outside will be reduced, might be completely wrong and the opposite might be true. Disturbing noises and conversations must be banned from medical treatments under these circumstances. However, it would be short-sighted to see the threat of injury only in negative terms. Negative is also the lack of positive suggestions. Earplugs that shield from disturbing noises and negative talks or attempts to avoid insufficient depth of anesthesia, however useful it may be, represent only the second-best solution. “Why are you giving a bolus of propofol right now?” I asked a resident giving general anesthesia. “I had the impression of insufficient anesthesia at that moment.” “Well, if that’s so, what would be the most important thing to do next?.” “?.” “To talk to the patient!.” It must be considered that probably the unconscious patient not only is the one who needs communication most, but whom it benefits most. After elimination or containment of negative stimuli the necessary second step is the realization that every opportunity must be utilized to support patients with therapeutic communication. It is indicated before, during and after stressful situations such as surgery, and regardless of whether the patient is awake or unconscious. Non-communication is hard for awake patients, for unconscious patients it is disastrous. The awake patient can satisfy his need for communication, the unconscious is left depending on grace and understanding of empathetic health care personnel.

The stressors reported by PTSD patients after accident, resuscitation, coma or intraoperative awareness are not pain or discomfort, as one might assume, but feeling alone, helpless, unable to draw attention to themselves, without control and being at the mercy of others, with the inability to communicate. The deficit is both from the patient’s side: “I was not able to express myself, they could not hear me.,” and from the health care side: “Nobody talked to me, they did not take notice of me.” It is a detrimental biological reflex that as soon as persons have their eyes closed, they are not recognized and addressed any more. Sedation is of no help but aggravates the situation.

Unconsciousness appears to represent an especially vulnerable phase, possibly because of lack of conscious safety mechanisms. Moreover, the lack of memories adds stress (Silva et al., 2019). Of course, early detection and post-trauma treatment of PTSD is indicated, although very time-consuming and often only partially effective (Peris et al., 2011; O'Toole et al., 2016). However, avoidance of stressful perceptions and influencing the stress during its generation and impact seems more important and more promising. Initial results on awake patients in an emergency department that showed less PTSD after life-threatening acute coronary syndrome associated with perceptions of good clinician–patient communication point in this direction (Chang et al., 2016). When we have lost consciousness, we may not remember, we may not react, but we may feel the presence of a caring person who speaks to us friendly and calmly, like a mother to her child (Silva et al., 2019). The solution for the communication deficit is no a drug, but communication. This is what the unconscious patient needs (Table 4).

**Table 4:** What is necessary for communication with unconscious patients?

Lower noise and avoid disturbing conversations.
Recognize and avoid negative signals and suggestions.
Not to continuously close down hearing with earplugs or music when this is the last channel to the surrounding and the patient is incapable of expressing himself.
Not to omit communication, but treat all as awake. This requires to override the natural reflex to stop communication when the other person has closed his eyes.
To announce treatments and manipulations by combining information with meaning.
To use hypnotic suggestions and communication to beneficially affect psychological and physical functions.

But what to say?

### 6.2 What to say to the unconscious?

In this regard, much can be learned from hypnosis, since not the conscious mind but getting in contact to and communicating with the unconscious mind lies in the middle of its expertise (Peter, 2024, this issue). The Hypnosis Definition Committee (HDC) of the American Psychological Association (APA) recently defined hypnosis as a “state of consciousness involving focused attention and reduced peripheral awareness characterized by an enhanced capacity for response to suggestion” (Elkins et al., 2015). Hypnotic trance can be defined as a non-ordinary state of consciousness that is accompanied by a number of neurophysiological changes, which can be detected directly by electroencephalogram and magnetoencephalogram and indirectly by near-infrared spectroscopy (NIRS), positron emission tomography (PET), and functional magnetic resonance imaging methods (Vanhaudenhuyse et al., 2014; Wolf et al., 2022; Miltner et al., 2024, this issue). Unfortunately, none of these methods has so far proven to be sufficiently specific for the characterization of the hypnotic state in general or for different degrees of the hypnotic state. Although individual parameters have repeatedly been claimed to be valid, they could not be confirmed by replications. Actually, this is not surprising given the diversity of hypnotic phenomena. Different brain activities are involved in motoric, sensory or cognitive tasks and effects. Moreover, while, for instance, most often hypnotic trance and hypnotherapy are associated with relaxation and “going deeper and deeper,” trance and the same hypnotic interventions and phenomena can be induced by active-alert hypnosis (Bányai, 2018). The latter is well known from sport-induced trance, and in part involves completely different brain areas. Some hypnotic phenomena now can be assigned or brought into connection to certain brain networks (Wolf et al., 2022). What used to be described with psychological terms such as “dissociation,” for instance, can be understood today as disconnectivity in the brain. As a whole, hypnosis represents the neurophysiological correlate of a subjective experience that presents itself as a sometimes extremely reduced self-reference, i.e., a lack of self-awareness, self-control or even a complete diminished self (Lynn et al., 2019; Peter, 2024, this issue).

Hypnosis can also be described as the skill to perpetuate and influence trance, be it induced (traditional hypnosis), or spontaneous. The latter results from the survival advantage of a natural trance as an emergency reaction providing effective skills of pain and stress control (e.g., dissociation) and access to physiological functions regulated in the unconscious (Cheek, 1962b; Hansen et al., 2024). Thereby, hypnosis can be effective without hypnotic trance induction in the form of “conversational hypnosis” (Short, 2018). Regardless of the trance induction, then hypnotic suggestions can be implanted directly into the patient’s unconscious, namely by the short-term elimination of conscious evaluation processes and the uncritical acceptance of the suggestions presented. Against this background, the content and also the form of the suggestions presented are decisive for the “unconscious” reception. Both forms of hypnosis have proven highly effective for many areas of medicine. Actually, strongest evidence for the use of hypnosis exists for acute medical interventions (Kekecs et al., 2014; Rosendahl et al., 2024, this issue).

Different biological, psychological, and social factors contribute more or less to outcomes in different subsets of individuals or for different conditions (Jensen et al., 2015). Little is known about the effectiveness of different hypnotic interventions when the brain is impaired by specific lesions, for instance those resulting in language deficits (left hemisphere). Examining the performance of a patient who suffered a stroke destroying most of his left hemisphere by two hypnotisability scales suggested that hypnosis can be mediated also by the right hemisphere alone A further study of 16 patients with unilateral strokes of the left or right hemisphere found no substantial differences in hypnotisability between the two groups (Kihlstrom et al., 2013). Psychotherapy is successfully applied in neurorehabilitation, yet without specificity to the brain lesions (Castelnuovo et al., 2016). In applications of hypnosis for awake craniotomies, i.e., brain surgery with awake patients, to avoid drug effects, no restrictions in the effects of hypnotic interventions were observed. This clinical experience concerns brain tumor surgery in the vicinity of eloquent or motoric areas (Hansen et al., 2013; Frati et al., 2019), and placement of electrodes for deep brain stimulation in Parkinson’s or tremor patients (Zech et al., 2018). Therefore, it appears neither possible nor necessary to select hypnotic interventions dependent on the detection of certain intact brain areas in brain injured patients. Above all, a specific neurological or neurophysiological diagnosis must not lead to the exclusion of the option for hypnotic therapeutic communication. In contrast, it is an argument of the “Conceptual Analysis” presented here that such exclusions from communication, for instance of unconscious patients, have been and are the origin of stress and further injury of patients. This concept does not aim to shape hypnotic interventions to a specific problem of a specific patient (like in hypnotherapy), or to the specific residual brain function capacity (after pharmacological, traumatic, or circulatory impairment). In contrast, it proposes to address basic psychological needs common to all patients, and to not limit provision of such communication to experts.

Both placebo effects and hypnotic interventions are based on meaning. Accordingly, it has been proposed to name the effect of conditioning and expectation “meaning response” instead of “placebo effect” (Moerman and Jonas, 2002). Similarly, hypnotic suggestions get their meaning and effectiveness through their meaningful content. But what is meaningful for the unconscious patient? It is the fulfillment of the basic physical and psychological needs. The following basic psychological needs have been identified (as outline by K. Grawe): Binding and affiliation, pleasure gain and displeasure avoidance, orientation and control, self-esteem enhancement and self-protection, and superordinated integrity and consistency (Grosse and Grawe, 2002). Their unsatisfaction leads to stress and trauma. The following stressors that have been identified in various groups with high risk for PTSD can be assigned to them: abandonment and not being able to express oneself, pain and suffering, chaos and futility, being at the mercy of others and hopelessness, degradation and threat, and superordinated disturbance and injury (Table 5). From there, 10 themes can be derived that should, or better, must be addressed in “a person in need,” be it a refugee, an accident victim, or a patient, conscious or unconscious: Accompaniment, contact, well-being, information, confidence, control, guidance, respect, safety, and healing (right column in Table 5). This principle has been used successfully to create texts for anesthesia induction (Hansen et al., 2024) or patients undergoing surgery under general anesthesia (Nowak et al., 2020), but moreover also allows to generate your own text for your patients (Hansen, 2024).

**Table 5:** Derivation of meaningful communication with persons in need.

Basic psychological needs	Stressors	Communication topics
BindingBelonging	Abandonment Impossible communication	**Accompaniment** **Contact**
Pleasure gainDispleasure avoidance	Pain, suffering	**Well-being**
OrientationControl	Chaos Futility Subjection Hopelessness	**Information** **Confidence** **Control** **Guidance**
Self-esteemSelf-protection	Degradation Threat	**Respect** **Safety**
Consistency, integrity	Disruption, injury	**Healing, order**

Important techniques of hypnosis in medicine include structured and controlled dissociation, as well as reframing of disturbing sensations (Hansen et al., 2024). Further principles for hypnotic communication are images of healing and the use of specific suggestions, such as cold, ice or snow to provoke analgesia and vasoconstriction. Other suggestions like the flowing of a stream target peristalsis or diuresis. The same applies to wound healing or immune responses (psycho-neuro-immunology) that are not regulated by reason and will, but by functional images anchored in the subconscious just like other involuntary bodily functions. Not relying on an alert mind, understanding and voluntary actions, and addressing the unconscious mind, hypnosis seems particularly suitable for patients where higher cerebral functions are temporarily impaired. Both placebo/nocebo responses and hypnotic suggestions can be understood as autosuggestion, in the sense of “communication to the subconscious” (Mommaerts and Devroey, 2012). Thus, the appropriate language for talking to unconscious patients is hypnotic communication, to “touch the unconscious in the unconscious.”

### 6.3 Significance for hypnosis and neuroscience

With hypnotic techniques adequate for handling patients in shock, during resuscitation, general anesthesia, or in coma, a wide field of application opens up in emergency medicine OR and ICU. This means a high demand for counseling and training of health care personnel that are close to the patients in these situations. The presented concept proposes use of hypnosis, however different from hypnotherapy without formal hypnotic induction. Furthermore, instead of a special therapy by a specialist for special patients, it proposes application of therapeutic communication of all health care staff to all patients, be they awake or unconscious (Hansen et al., 2024). An unexpected and remarkable result of the aforementioned study published in BMJ (Nowak et al., 2020) was equal or better effectiveness of suggestions during general anesthesia compared to hypnosis in wake patients (Kekecs et al., 2014), with effect sizes of 0.45 vs. 0.35 with regard to pain reduction, and 0.36 vs. 0.23 with regard to the reduced need for analgesics, respectively. One of the strongest evidence for the use of hypnosis exists for medical interventions (Rosendahl et al., 2024, this issue). The results and correlations discussed in this Conceptual Analysis can be a stimulation to extend hypnosis for patients undergoing surgery from pre- and post-operative application to include also the intraoperative phase. This extends the communicative intervention thus from prophylaxis and therapy to prevention of stress and psychological trauma by including the time of the traumatizing event. We have to consider, for instance, that the unconscious mind realizes when the own heart stops beating, be it in heart surgery or resuscitation, and that it is an unimaginable threat to experience your own cardiac arrest, and most probably traumatizing – when it is not accompanied by communication.

Hypnotherapy utilizes a state of consciousness modified by induction of trance, where the critical mind with its filter function is suppressed and the effects of suggestions are enhanced. Actually, bypassing normal consciousness and thinking seem to be essential features of hypnosis to allow access to the unconscious and responses to “suggestions” (in the sense of the Latin meaning “to slide underneath”). Hypnosis has to do with the induced loss of the sense of agency (SoA), the sense of self, and with the experience of involuntariness in the induced responses (Peter, 2024, in this issue). These characteristic features are also found in the discussed disorders of consciousness. Moreover, major aspects influencing consciousness such as attention, perception, cognition, or memory can be impaired in those states of unconsciousness. On the other hand, those are aspects that can be precisely influenced by hypnosis via modulation of brain structures involved in the regulation of consciousness, and via use of altered brain activities for increased capacity to respond to suggestions. Accordingly, hypnosis can be utilized to elucidate unconscious processing, somehow like a vehicle to uncover the unconscious mind (Landry et al., 2014). By specifically attenuating certain brain areas and their connections, for instance by dissociation, it also can serve for models of brain damage. Similarly, pharmacological hypnosis (called general anesthesia) is a probe to explore consciousness and its disorders (Mashour, 2013; Bonhomme et al., 2019). A meta-analysis of 36 studies about functional imaging, namely fMRI, PET and SPECT, in patients with DoC (mainly after traumatic or anoxic brain injury) consistently revealed markedly reduced activity in anatomic structures that have been linked to the default-mode-network (DMN) (Hannawi et al., 2015). Precisely modulation of this network has been identified as a neurophysiological basis of hypnosis as well as of loss of awareness (Demertzi et al., 2011; Walsh et al., 2017). Deactivation of the DMN, for instance, correlates with the subjectively perceived depth of hypnosis (Peter, 2024, this issue).

In conclusion, hypnotic communication and interventions in patients with coma or other disorders of consciousness including cardio-circulatory arrest and general anesthesia have the potential to mutually stimulate and enrich research on consciousness, coma and hypnosis. From disorders of consciousness, from drug effects on brain functions, and from hypnosis we can learn about the human brain and about the condition we call consciousness. Hypnosis provides a tool with effects on both the level of consciousness and its specific components including attention, dissociation, and memory. Future research should of course evaluate clinical, psychological and physical effects of such communication with unconscious patients. Effects can be expected on stress parameters, on side effects like pain or nausea, on homeostasis, and on healing progress, as well as the incidence of psychological sequelae like delirium or PTSD. Further research should include analysis of brain-specific biomarkers (tau, NfL, GFAP, UCH-L1, etc.) as physical consequences of the intervention “hypnosis” on an impaired brain. To strengthen and support the proposal for a general communication with unconscious patients, further evidence for perception under these medical situations, e.g., by monitoring during, and by establishment of structured interviews after brain damage, resuscitation, general anesthesia, and intensive care, would be helpful. However, tests for responsiveness should not further be limited to nociceptive triggers, sounds or neutral signals, but include meaningful communication, because meaning seems to be a major determinator of unconscious perception and resulting responses.

## Author contributions

EH: Writing – original draft, Writing – review & editing.
